# How food systems change (or not): governance implications for system transformation processes

**DOI:** 10.1007/s12571-021-01178-4

**Published:** 2021-06-18

**Authors:** Cees Leeuwis, Birgit K. Boogaard, Kwesi Atta-Krah

**Affiliations:** 1 Knowledge Technology and Innovation (KTI), Wageningen University, Wageningen, the Netherlands; 2 Director Country and Regional Engagement, International Institute of Tropical Agriculture (IITA), PMB 5320, Ibadan, Nigeria

**Keywords:** Governance, Policy recommendations, Food systems transformation, Systems thinking, Intervention, Transition

## Abstract

This paper argues that supporting food system transformation requires more than obtaining science-based understanding and analysis of how components in the system interact. We argue that changing the emergent properties of food systems (what we call food system synthesis) is a socio-political challenge that is affected by competing views regarding system boundaries and purposes, and limited possibilities for central steering and control. We point to different traditions of ‘systems thinking’ that each emphasize particular types of interventions for achieving system change, and argue that food systems are best looked at as complex multi-dimensional systems. This implies that we need to move beyond rational engineering approaches to system change, and look for approaches that anticipate and accommodate inherent social tensions and struggles in processes of changing food system dynamics and outcomes. Through a case study on the persistence of an undesired emergent property of food systems (i.e. poverty) we demonstrate that a multi-level perspective (MLP) on system transformation is useful in understanding both how food system transformation has happened in the past, and how desirable transformations is prevented from happening today. Based on such insights we point to key governance strategies and principles that may be used to influence food system transformation as a non-linear and long-term process of competition, negotiation and reconfiguration. Such strategies include the creation and nurturing of diversity in the system, as well as process interventions aimed at visioning, destabilization and formation of discourse coalitions. Such governance interventions imply a considerable re-orientation of investments in food system transformation as well as a rethinking of the role that policy-makers may play in either altering or reproducing undesirable system outcomes.

## Introduction

There is currently a widespread interest in the idea of transforming food systems as a strategy to realize a variety of development objectives, including health, inclusiveness, safety, sustainability, efficiency and resilience (HISSER). Food systems are conceptualised as complex wholes in which a variety of human and non-human drivers interact with activities in agro-food value chains, resulting in desirable or undesirable outcomes (see e.g. HLPE, 2017; Van Berkum et al., 2018). We currently witness several efforts to develop systematic frameworks for the analysis of food systems (Foran et al., 2014; Gaitán-Cremaschi et al., 2019; Hanna et al., 2020; Kanter et al., 2015; Termeer et al., 2018; Zurek et al., 2018). It is assumed that such analysis can increase our understanding of the way in which components in the system interact, and provide insight in trade-offs and synergies between development objectives associated with alternative interventions in the system. Thus, food systems analysis is seen to offer opportunities for making foresight scenarios and ex-ante impact assessments that can guide decision-making on innovation and investments strategies to support the realisation of desired multi-dimensional outcomes (see HLPE, 2017).

The starting point for this chapter is that generating detailed knowledge and understanding about food system dynamics and the likely positive or negative consequences of alternative courses of intervention does not in itself bring about food system transformation. Assuming that it does, would be the same as assuming that farmers’ adoption of innovations depends only on whether they have appropriate knowledge and understanding of alternative options. Clearly this is not the case; we know from sociological and psychological research and theory that such knowledge and understanding is only one out of many factors influencing what people do and do not do. In the context over governance, knowing about the potentially transformative effects of alternative policies and measures implies by no means that such changes will be agreed upon and/or can be successfully implemented to alter food system dynamics.

In this chapter we will first outline some challenges that exist in the governance of food system transformation. In doing so, we will make clear that ‘food systems synthesis’ (putting together a newly adapted food system that produces desirable outcomes) requires more than ‘food systems analysis’ (developing refined understanding of how different parts of the food system function and interact with each other). Subsequently, we present what is already known about the way in which complex socio-technical systems transform over time based on historical precedents. Finally, we will discuss the implications for governing food system transformations in terms of the types of interventions that are needed to make systems change.

## Six relevant features to consider when thinking about food system transformation

**Fig. 1:**
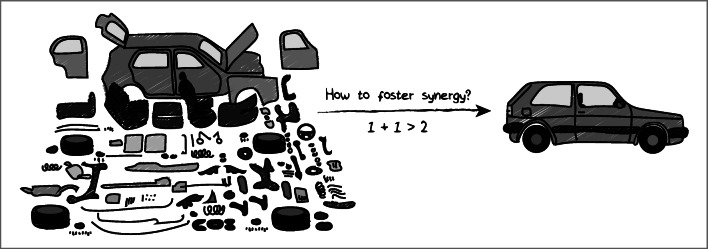
Emergent properties and synergy: ‘the whole is more than the sum of the parts’ (source: own elaboration)

When considering how we may – or may not - transform food systems, it is useful to think about some pertinent features of these kinds of systems that affect our capacity to transform and reconfigure them. Systems thinking is usually applied in situations where we deal with complex wholes that comprise of multiple entities, processes and interactions, that result in a range of outcomes which we may consider more and less desirable (Meadows, 2009). A first and fundamental feature of systems is that they can exhibit ‘emergent properties’, which refers to idea that ‘the whole is more than the sum of the parts’. For example: a car consists of many parts (an engine, gearbox, doors, wheels, chairs, driver, etc) and when these are put together in a proper way, we end up with a system (car) that can drive from A to B, which is a property that none of the individual parts has (Fig. 1). When we like the emergent property of the system, we tend to speak of ‘synergy’ (1 + 1 > 2) and if we do not like it we may speak of a ‘negative synergy’ (2 + 2 < 4) or ‘disergy’. From this perspective, the interest in ‘food system transformation’ reflects that we are not very happy with the ‘emergent properties’ (or the trade-off between positive and negative synergies) of current food system configurations: instead of desired properties such as ‘healthy nutrition’, ‘food security’, ‘wealth’ and ‘environmental sustainability’, we see that our food systems still generate ‘malnutrition’, ‘food insecurity’, ‘poverty’ and ‘environmental degradation’ (Béné et al., 2019; Veldhuizen et al., 2019; Willett et al., 2019). These *emergent properties* thus constitute a first relevant feature of food systems.

A second important feature of food systems is that - unlike the example in Fig. 1 - *interactions between people are essential components* in the system. Most boundary definitions of food systems include actors that operate at different levels and in different spheres, where different ‘emergent properties’ may be seen as relevant (see also Van Berkum et al., 2018; Van Berkum et al.*, this volume). Typically, we are dealing with entities, processes, interactions and outcomes at the level of households, farms, communities, natural resources, trade networks, processors, retail, consumers, policy, climate, and what may be considered beneficial at one level or sphere (e.g. low consumer prices, high farm productivity) can be seen as less desirable when seen from another perspective (e.g. low margins for producers, environmental degradation). Moreover, any interventions in the system to influence a particular benefit for people at one level, is likely to go at the cost of other values at the same or other levels. It is precisely these complexities and trade-offs that merit a systems perspective, but it is by no means self-evident what and whose priorities at what level should prevail in a discussion about transformation.

Third, it is important to realize that *food systems are diverse* (Gaitán-Cremaschi et al., 2019). Even within the same geographical space and/or value chain, we see that there exist different segments and networks of producers, traders, processors, retailers and consumers that work in different ways (Mangnus, 2015; Mangnus & Vellema, 2019), and with different levels and forms of formality, quality control, etc. The networks and processes involved in supplying palm oil to rural consumers in Ghana are likely to be very different to those supplying urban elites in Accra or European consumers. This diversity means that there is not just one food system that operates according to one particular logic, but that there exist multiple parallel systems that serve different producers and consumers.

**Fig. 2:**
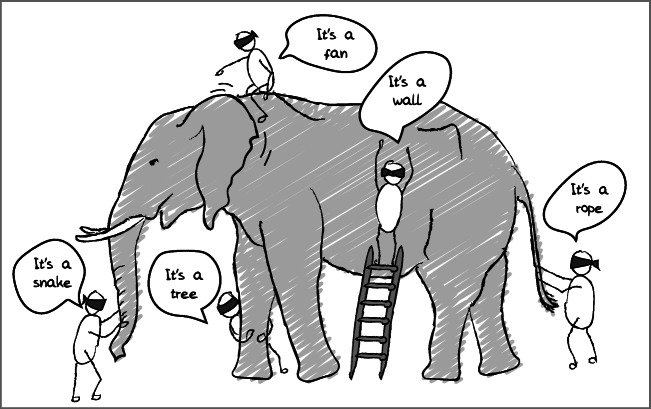
Different actors do not have the same conception of the system (source: ‘The blind men and the elephant’, inspired by an Indian parable)

There is not only diversity between food systems, but also *diversity between actors* in the way they view the system and the different purposes and interests they have for participating in it (Fig. 2). People in the system are not likely to perceive the system in the same way as those who observe and analyse the system from a distance. Most actors in the system are likely to have only a partial and selective view of it, and they may define the system in completely different ways. To illustrate this fourth relevant feature of food systems: some may see a farm as ‘a production unit that is part of a food supply chain’ whereas the person actually farming on it may see it as ‘a last livelihood resort’, or as ‘a family land that needs to be maintained to please the ancestors’, or as ‘a community resource that guarantees independence from the outside world’. Similarly, where some may look at informal trade ‘a sub-optimal process that jeopardizes food quality and food safety’, others experience it as ‘a reliable network to ensure access to markets and credit’. Such differences in perspectives are likely to complicate the fostering of synergy and emergence in a particular direction.

**Fig. 3:**
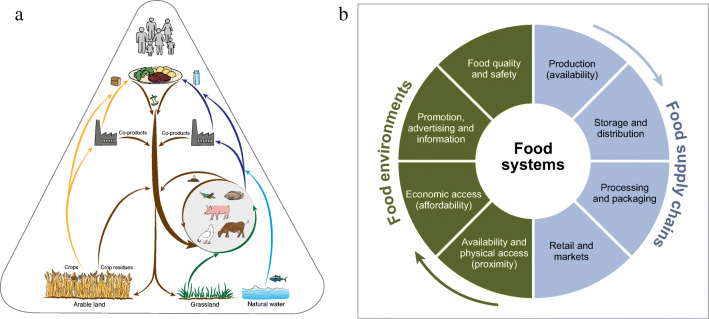
Two different ways of conceptualising a food system. **a**. The role of farm animals in a circular food system (Van Zanten et al., 2019). **b**. Food supply chains and food environments (adapted wheel concept from Ranganathan et al., 2016 in HLPE, 2017)

In this line, it is important to note there exist different ways of conceptualising food systems among researchers. In general, researchers tend to subscribe to different theoretical ideas regarding the nature of systems, the process through which systems change, and the kinds of interventions that may be appropriate to support transformation (see e.g. Hanna et al., 2020). This also counts for food systems, where we see that scientists may have different conceptions of the key entities, processes and outcomes that constitute a food system. For example, a food system can be conceptualized to study the role of farm animals in a circular food system (Fig. 3a) or to study food supply chains and food environment, in terms of food quality and safety, affordability, and availability (Fig. 3b).

These two examples underline that systems do not exist as an unequivocal objective reality, but that they are ‘constructs’ that we apply to the world in order to make sense of a complex phenomenon, based on particular epistemological positions - i.e. how we know about the world (Armson, 2011). Over time, different strands of systems thinking have developed, such as hard systems thinking, functionalist systems thinking, soft systems thinking, and complex system thinking (Table 1). Different strands of thinking about systems are relevant to different kinds of problem situations - varying from simple to complex – each with specific epistemological foundations. Awareness of underlying epistemologies is especially important in messy situations, like food system transformation, where people may have a very different view of the same situation (Armson, 2011).

**Table 1:** Different strands of systems thinking (adapted from: Leeuwis & Wigboldus, 2017; see also Leeuwis, 2004)

Type of systems thinking (origin and/or literature sources)	Key metaphor and assumption depicting how systems are seen	Key change strategy implied	Example in a food system context
Hard system thinking (scientific management, Taylor, 1947)	Machines Interactions in natural and social systems can be known and predicted	Engineer and optimize towards a given goal	Use crop growth models to decide where and how crops should be grown in order to maximize efficiency or minimize risk.
Functionalist systems thinking (human relations management, Roethlisberger & Dickson, 1961; structural functionalism, Parsons, 1951)	Organisms Systems are functional wholes, depending on relations between components and environment	Re-balance and adapt in a changing environment	Increase the weight of ‘drought tolerance’ as a criterion for variety selection in response to climate change.
Soft systems thinking (Checkland, 1981; Churchman, 1979)	Meanings Systems consist of people with different worldviews and boundary definitions	Foster dialogue, learning and agreement among actors	Bring all stakeholders in a food system together to exchange goals and views in the hope that they reach agreement on a way forward.
Cognitive/Autopoietic systems thinking (Luhmann, 1984; Maturana & Varela, 1984)	Psychic prisons Biological and social systems tend to perceive the world through their own logic and be blind to others	Shock therapy by creating a crisis	Threaten stakeholders in a food system that draconic measures will be taken unless they come up with an agreed upon plan at a certain deadline.
Political/Critical systems thinking (Jackson, 1985; Ulrich, 1988)	Arenas of struggle Systems are characterized by power structures that constrain system change	Coalition building, competition and negotiation	Bring together those actors in a food system that are in favour of biological pest control, and provide them with resources to lobby against the pesticide industry.
Social/Institutional systems thinking (Giddens, 1984; North, 1990)	Rules Formal and informal rules are produced and reproduced in interaction, resulting in certain orders	Change rules and incentive structures	Combat obesity by introducing a progressive sugar tax for food processers and retailers that sell sugared foods.
Complex systems thinking (Prigogine & Stengers, 1984)	Self-organisation New orders emerge without central steering as the unplanned result of multiple intentional actions	Identify existing trends and opportunities arising from these	Install a multi-disciplinary foresight committee to identify simultaneously occurring trends and pressures in food systems, and discuss what latent futures may become more feasible.

Food systems thus include many stakeholders with different views and interests. In addition, the interactions between actors are guided by formal and informal institutions (i.e. ‘rules of the game’). Prevailing legal, market and cultural rules and resource distributions tend to have a degree of stability, not least since attempts to change the status quo are likely to be resisted by those who benefit from the existing system configuration. Frequently, these include powerful groups that can mobilise considerable resources to prevent change from happening (Grin, 2012). In addition, the scope for system transformation may be hampered by path dependencies, whereby earlier choices (e.g. choices to support certain crops, or decisions to invest in specific technologies or organisational set-ups) make it difficult to deviate from a chosen path (Kuokkanen et al., 2017). Moreover, the fact that food systems include so many public and private players and stakeholders with different views and ways of working implies that -in most cases- there is no actor that has sufficient power and control to steer the system in a particular direction. Food systems are thus characterized by high uncertainty with no – or hardly any – agreement on the goals, values and boundaries that should drive and direct change. Such complex configurations do not have a central locus from which a system is or can be steered and controlled. Instead, numerous actors and organizations pursue their own goals and projects, and the resulting changes are largely the unintended outcome of numerous intentional actions which interact and interfere with each other in complex ways (Castells, 2004; Scharpf, 1978; Van Woerkum et al., 2011). This is a fifth relevant feature of food systems, called *self-organization*, which refers to the evolution and emergence of new patterns and orders without central steering and control (Leeuwis & Aarts, 2011; Nicolis, 1989). Box 1 provides an example of self-organisational dynamics in food systems.

**Table:** Box 1 An example of self-organisation: The emergence of an obesogenic pattern in food systems

**The emergence of an obesogenic pattern in food systems**	
In many societies obesity has become an increasing health problem that is clearly related to the functioning of food systems. Thus, overweight can be seen as an emergent property of food systems. According to the literature (see e.g. Swinburn et al., 2011), the obesogenic pattern has emerged from the interaction between several relatively autonomous trends. These include: (a) increased capacity to produce cheap processed foods; (b) wide proliferation of outlets and marketing for cheap nutrient poor foods with added sugars and unhealthy fats; (c) changes in the type of work we do (non-physical labour); (d) changes in the pace at which work is done (stress); (e) changes in our transport systems (from bike to car); (f) changes in the way cities are designed (conducive to motorized transport); (g) the increased importance of ICT in work, leisure and education; etc. Even if some of these trends may have resulted from intentional activity (e.g. creating accessible cities, developing computer games that are fun, increasing the sales of sugared food, etc.) none of these developments can be associated with a deliberate ambition or plan to foster an obesogenic society. Thus, the obesogenic pattern emerged in a self-organised manner: that is, as an unintended outcome of many intentional actions geared towards something else. In the case of obesity, this unintended outcome is nowadays apparent to most actors involved. Even so, several actors can be seen to deliberately continue their practices (e.g. selling fat and sugared foods in vulnerable neighbourhoods) in order to pursue their priority goals (e.g. making profit). Thus, they ignore system feedback and wittingly reproduce the emergent outcomes.	

Hence, food systems are complex, diverse and self-organizing wholes in which relatively autonomous stakeholders have competing interests, values and perspectives, and where transformation depends to a considerable extent on the willingness and capacity of interdependent actors to accommodate and navigate differences and work towards a mutually acceptable future. This means that food systems are not easily transformed. This is not to say that food systems are static; in fact, they are characterised by continuous dynamism, interaction and flux. At the same time, they tend towards a relatively stable pattern of interactions and outcomes (i.e. earlier mentioned emergent properties) and are often resilient to efforts to change them and/or and slow in responding to internal or external pressures and shocks. Such ‘*dynamic stability’* is a sixth key feature of food systems and is characteristic for complex problems (Arkesteijn et al., 2015). Because stakeholders are interdependent, meaningful change is likely to happen only if key players succeed in achieving a sufficient degree of agreement, accommodation and/or coordination to work towards a particular transformation, and in translating this towards new policies and institutional arrangements. Reaching such convergence can be complicated in various ways, for example by lack of interaction and facilitation, and also by the existence of uncertainty about how proposed changes may affect system dynamics and outcomes. Such lack of knowledge and understanding typically reinforces people’s perception that the risks associated with change are high. At the same time, it is important to acknowledge that food systems are not isolated from other systems, and that crisis and shocks in other realms (e.g. Covid-19, Ebola, war) may put so much pressure on food systems that interdependent stakeholders can achieve accommodation towards meaningful change.

Summarizing, the following six key features of food systems need to be taken into account when thinking about food system transformation: (1) emergent properties of a food system, (2) human activities and interactions as central components, (3) diversity between and within food systems, (4) diversity between actors’ views, values and interests, (5) self-organizational dynamics in food systems, and (6) dynamic stability and resilience of food systems. Based on these key features, it can be concluded that food systems are best looked at as complex multi-dimensional systems, and that efforts to transform food systems in particular directions - i.e. *food system synthesis* - need to grapple with issues proposed by different strands of system thinking (Table 1), including especially political/critical systems thinking, social/institutional systems thinking, and complex systems thinking (Table 1). In a process of food system synthesis, interdependent stakeholders can -to some degree - resolve their differences, build conducive relationships and overlapping visions on the future. This goes far beyond an analytical exercise geared towards understanding (parts of) the system, and using this technical and/or social understanding to design and optimise the system through some kind of rational engineering logic. This is bound to fail as it ignores the inherent social tensions in the system such as different boundary definitions, diverging perspectives, competing interests, different epistemological foundations, and dynamics of power. In a process of *synthesis* such differences and tensions need to be accommodated, which requires broader processes than developing analytical understanding.

## How transformation happened (or not) in the past: Understanding the persistence of poverty^1^

It is generally acknowledged that the existing dominant food system generates problematic, undesired and often unplanned consequences such as environmental degradation, economic exploitation, malnutrition, food insecurity, increased inequalities, and poverty (McMichael, 2013). As mentioned in the previous section, transformation in food systems can be seen as a governance effort to alter such undesired emergent properties of the system into desired properties such as ‘healthy nutrition’, ‘food security’, ‘wealth’ and ‘environmental sustainability’. Although it is generally agreed that a transformation is needed to move towards more sustainable food systems across the globe, development programs over the past decades have not been very successful in fundamentally changing the food system towards more sustainable systems. When we zoom in on poverty as an emergent property of current food systems, we see that – even though the percentage of extreme poverty in the world has reduced in the past decades – poverty reduction is slowing down or has reversed in many countries, and remains at an unacceptable high level, especially in sub-Saharan Africa (World Bank, 2019). This despite the fact that poverty reduction is one of the key objectives of programmes that invest in transformation of food systems (see e.g. CGIAR Consortium Office, 2015).

We will explore major historical insights on how systems transformation has happened or been prevented in the past. We will draw on the evidence-based multi-level perspective model (MLP) on transformation processes that has been developed by historians (Geels & Schot, 2007) and that has also been applied to understand change in food systems and agriculture (see e.g. El Bilali, 2019; Gaitán-Cremaschi et al., 2019; Gaitán-Cremaschi et al., 2020; Hinrichs, 2014; Meynard et al., 2017). It will be argued that many fundamental transformations in the world have happened in this manner and – as we will show –occurred rather unplanned (Geels, 2011). Through a case study on the persistence of poverty we show the complexity of food system transformation. In other words; why does poverty persist despite the fact that numerous interventions and governance efforts in food systems are geared towards reducing it? Why is it so difficult to change poverty?

### How change happens according to the MLP model

The MLP model is different from the frequently used adoption/diffusion theories on innovation (see e.g. Rogers, 1995) in the sense that it is based on analyses of historical transformation processes, which in MLP are labelled ‘system innovations’. Based on evidence from historical case studies – such as the American transition from horse-drawn carriages to automobiles (Geels, 2005) and the British transition from sailing ships to steamships (Geels, 2002) – the MLP model gives insight in how complex socio-technical systems transformed over time. The MLP model has been widely applied to agriculture and food systems, and is particularly useful to gain a better understanding of sustainability transitions such as food systems transformation and poverty reduction, because these are socio-technical transformations characterized by long-term processes with multiple actors in complex institutional settings (Geels, 2011).

**Fig. 4:**
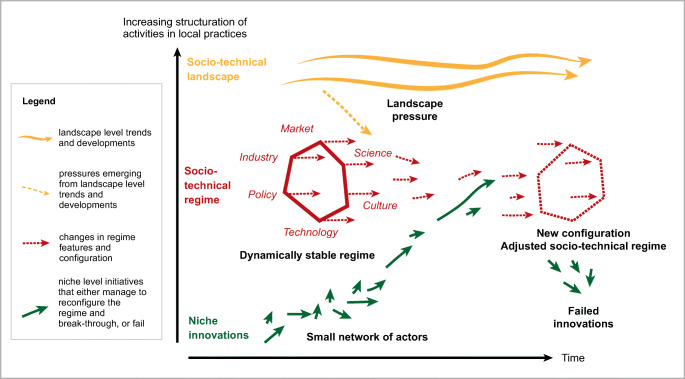
The multi-level perspective on system innovation (adapted from Schot & Geels, 2008)

A central notion of the MLP model is the fact that system transformation results from the interaction between dynamics at three levels: niche, regime and landscape (Fig. 4, adapted from Schot & Geels, 2008). *The socio-technical regime* is built up by multiple interrelated domains, such as policy, industry, technology, markets, science, culture (red in Fig. 4). These domains form the ‘deep structure’ that provides a certain stability to an existing system, for example the fossil fuel based energy system that has been dominant for the past 100 years or so, and is only recently being challenged seriously. The activities of social groups in these different domains (in the fossil fuel example: car manufacturers, designers of combustion engines, oil industry, filling stations, automotive dealers, advertising companies, etc.) are coordinated in such a way that the socio-technical regime is reproduced over time. Thus, one can say that there is a set of semi-coherent rules and relations that keep the socio-technical regime in place (Geels, 2004, 2011). MLP starts from the premise that a transformation occurs when regime dimensions are reconfigured (red dotted lines in Fig. 4). For food system transformation this means that the current food system^2^ is embedded in a specific socio-technical regime, which needs to change. Before we explore why this is inherently difficult, we first look at niche level.

*Niche innovations* form the micro-level where radical novelties emerge (green arrows in Fig. 4). Niches come from outside the dominant socio-technical regime, and are aimed at transforming the existing regime. In the energy example we can think of initiatives that develop and promote renewable energy (e.g. solar energy, wind energy, hydrogen, biofuels) and associated transportation systems. Niches are essential to create change and can be seen as experimental spaces where new ideas, technologies, organizational structures, etc. are formed, piloted and become gradually mature, i.e. “they provide the seeds for systemic change” (Geels, 2011). Niche innovations thus try to challenge, disrupt, transform and break through the existing regime (green arrows in Fig. 4). Due to the dominance of the existing socio-technical regime, it is difficult for niches to break through, which is one of the main reasons why new innovations may fail (see failed innovations in Fig. 4). The MLP model thus shows that in complex socio-technical transformation processes there are no quick fixes, but it requires long-term deep-structural changes at regime level (Geels, 2011). Change thus happens gradually with modifications in technology as well as in cultural, political, scientific, market and industrial domains (Geels, 2011). Therefore, niches need to “build up internal momentum, through learning processes, price/performance improvements, and support from powerful groups” (Geels, 2011; Geels & Schot, 2007). Transformations can be hindered because niche innovations may not be ready yet or because the interaction between regime and niche is antagonistic. As such, niches are sometimes seen as ‘incubation rooms’, where novelties need to be protected against mainstream market selection in order to become successful (Geels & Schot, 2007). For example, organic agriculture can be seen as a niche that tried – and continues to try – to alter the current dominant conventional food regime (e.g. Metelerkamp et al., 2020).

*The socio-technical landscape* is the dynamic exogenous environment that is beyond the direct influence of actors at niche and regime level (Geels & Schot, 2007) where developments take place that put pressure on the existing regime and/or that creates new opportunities for change (yellow in Fig. 4). One can think for example about macro-economic developments (e.g. the financial crisis), population dynamics (e.g. population growth), and macro-political developments (e.g. international tension and conflict). The emergence of these changes can be gradual or sudden in the form of a shock. For example, climate change has been a slow process that has resulted in a gradual pressure on food systems, whereas the current Covid19 pandemic is a rapid external shock at landscape level that has consequences beyond the bio-medical domain and can result in an immediate crisis (with long term implications) in food systems (Clapp & Moseley, 2020; van der Ploeg, 2020).

Summarizing, whether such socio-technical transformation occurs depends on multiple factors (Geels & Schot, 2007), including: (a) whether niches exist and/or are ready; (b) whether interaction between niche and regime is cooperative or antagonistic; and (c) whether landscape pressures on the system are sudden or gradual. From the above follows that part of the difficulty of changing an existing regime, is that it is characterized by a certain lock-in (i.e. a difficulty to escape from dominant technical and social arrangements in the regime) that hampers the progression and survival of developments at niche level. A regime is rather ‘stable’, in the sense that elements of a socio-technical system are reproduced with slight adaptations, while the architecture remains unchanged – the existing regime is thus not ‘static’ but ‘dynamically stable’. Thus, in addition to helping us to understand how change can happen, MLP also clarifies how change may be obstructed: an existing regime continues to exist without a breakthrough of a new configuration.

### Why poverty persists: Resilience of the existing socio-technical regime

As indicated earlier, poverty is an emergent property of the current food system, and from the above elaboration of MLP we can learn that this system is embedded in a socio-technical regime. To better understand the persistence of poverty, it is thus important to understand the mechanisms that keep the existing socio-technical regime in place. To start with, it is important to recognize that socio-technical transformation processes are shaped by different actors who interact, struggle, form coalitions, and negotiate space in order to create change (Geels, 2011; Geels & Schot, 2007; Ramos-Mejía et al., 2018). Transformation processes are thus about politics and power struggles between actors at regime and niche level (Geels, 2011). Dominant players often try to maintain the current situation, which leads to a certain ‘lock-in’ of the existing regime. The development of strategies to overcome such constraints may benefit from a better understanding of the dominant configuration. Thus, relevant questions are: What are characteristics of the existing regime? What rules and actors dominate and maintain the current regime?

The socio-technical regime that dominates current global food system is a largely neo-liberal, capitalist, market-led and corporate regime based on models of economic growth (McMichael, 2016). This regime is shaped by multiple domains, such as policy, industry, technology, markets, science, culture. For example, the dominant industry consists of large scale, (inter)national, corporate businesses, which are largely oriented to maximizing profit and shareholder value, with less emphasis to other values (such as poverty alleviation or environmental sustainability). Similarly, the dominant trade policy focuses on international and national formal markets and value chains, and the dominant political doctrine is neo-liberal, with the state largely facilitating private business and competition in open markets, rather than protecting local economic systems that cannot compete with global players (McMichael, 2016). Agriculture, food, and development are then mainly seen as governed by market relations, whereby inequality becomes accepted as a logical consequence of market rationality (Da Costa & Mcmichael, 2007). For example, the availability and use of cheap agricultural labour is beneficial to producing sufficient and affordable food in a capitalist society, and dominant players see little advantage in redressing this, even if it arguably maintains poverty (Da Costa & Mcmichael, 2007; McMichael, 2016). Power is unequally distributed along value chains as “markets by their very nature tend to either exclude or exploit those with little power” (Kabeer, 2004 in Green & Hulme, 2005: p875). Market institutions and formal market logic thus deepen inequalities (Da Costa & Mcmichael, 2007).

This dominant regime has been widely criticized and we do not aim to repeat all criticisms here (see e.g. Da Costa & Mcmichael, 2007; McMichael, 2015; van der Ploeg, 2009). What is relevant for the current paper is to realize that transformation processes are arenas of struggle and negotiation and that not all actors have an equal voice. For example, smallholder farmers can be given less voice and space at the negotiation table compared to actors of dominant capitalist-oriented system. Thus, certain actors are included, whereas other are excluded (Klerkx & Rose, 2020). This not only accounts for the actors, but also for their epistemologies. For example, evidence from a study on organic agriculture in South Africa showed that niche innovations on organic agriculture hardly break through the existing food regime (Metelerkamp et al., 2020). The existing regime is largely based on mainstream Western scientific knowledge, in which other forms of knowledge are excluded. Knowledge on organic agriculture is hardly taught in conventional agricultural education in South Africa and as such “established institutions may currently do more to perpetuate the current food regime than they do to support emerging alternatives” (Metelerkamp et al., 2020). The same can be said concerning agroecology or agroecological agriculture. Consequently, marginalized knowledge easily remains marginalized unless there is a willingness of actors in the existing regime to change fundamentally. Such an urge for change may occur through development at the landscape level which put pressure on the socio-technical regime. For example, the Covid19 pandemic puts severe pressure on dominant socio-technical regime of the current global food system, reinforcing calls for radically different options, such as the proposal “to take agroecology as the ordering principle for agricultural production worldwide” (Van der Ploeg, 2020: p21). However, reconfiguration is inherently difficult because poor, marginalized and exploited groups of people and their knowledge tend to be excluded.

### Planned interventions have limited influence

There have been numerous projects and programmes that aimed at creating a system transformation with regard to poverty^3^ including many programmes that focussed on dimensions of the food system. Cobbinah et al. (2013) mention five periods with specific poverty interventions in the Global South, largely led by the Global North. In short: the 1950s and 1960s were characterized by capital investments, mainly in infrastructure. In the 1970s, there was more emphasis on social investment, including health and education. While in the 1980s the World Bank led a two-part strategy by 1) promoting labour-intensive growth through economic openness and investment, and 2) providing basic services. In the 1990s, the Poverty Reduction Strategy Papers (PRSP) by the World Bank focused on governance, social institutional frameworks and community participation. From 2000 onwards poverty reduction is part of the MDG’s and currently the SDG’s, in which it is approached as a rights-based issue (Cobbinah et al., 2013). It should be noted here that there is significant debate on how poverty should be defined and measured (L’Huillier, 2016). While there is evidence that percentages of absolute poverty have reduced in the past decades (World Bank, 2019) - e.g. through the strengthening of safety nets (Monchuk, 2013) -, others propose that poverty needs to be looked at more broadly, and take into account inequalities of poor, marginalized, and exploited groups with regard to e.g. health, education, basic needs, living environment, rights, and gender relations (Cobbinah et al., 2013; Green & Hulme, 2005). From this latter perspective, it can be concluded that poverty has persisted despite decades of intervention in food systems (Cobbinah et al., 2013; Green & Hulme, 2005) and that many aid interventions failed to bring about transformative change (Easterly, 2006; Moyo, 2009; White, 2001). The MLP model can help to understand why planned interventions by the national and international community have had limited success.

The above-described poverty interventions can be described as ‘mainstream’ type interventions. Most of these types of interventions are in line with the existing regime in that they build on market-based logics, and as such *reproduce the existing regime* including its tendency to maintain poverty. Thus, such poverty interventions are likely to strengthen the existing dominant regime rather than challenging it, thus reproducing mechanisms that are known to foster poverty (Da Costa & Mcmichael, 2007). In fact, planned interventions can even *worsen* the situation. For example, Nieusma and Riley (2010) demonstrated how development projects tend to over-focus on technologies, thereby occluding and even increasing social injustices and inequalities. Likewise, a study by Ramos-Mejía et al. (2018) provides evidence that socio-technical transformations tend to reproduce poverty patterns and worsen inequalities, in the sense that existing regimes strengthen the privilege of a few, while undermining the well-being of many. Similar phenomena have been observed around agricultural projects, where scaling of technology has been accompanied with scaling of exclusion and marginalisation (see e.g. Bouwman et al., 2020; De Roo et al., 2019). This puts the issue of politics, inequalities, and power struggles at the center of system transformation processes. Such struggles are all the more relevant in poverty contexts, because these contexts are often characterized by deep inequalities (Ramos-Mejía et al., 2018). It means that power imbalances may need to be reconfigured in order to enhance a transformation (Ramos-Mejía et al., 2018).

Although there is a diversity of ‘mainstream’ interventions and programs, the general narrative behind these interventions is that of changing the world through technology and planned interventions. This mainstream view on progress refers to what Jasanoff (2002) describes as “a single grand narrative” of the past 50 years: technology and science lead to progress and growth. However, over the past decades it became clear that “the material world cannot be governed and manipulated in new ways without also profoundly reordering society” (Jasanoff, 2002: p255), which includes societal changes in terms of power relations as well as changes in views and perspectives on e.g. food systems. This is also shown in the MLP model: transformation does not occur only through an adjustment in technology but requires a reconfiguration of the existing regime so that an enabling environment is created in which marginalized groups can benefit from technology. This may require radical changes in several regime dimensions, including for example: markets rules and international trade regulations that provide incentives to marginalised producers (Koning, 2017; Koning & Pinstrup-Andersen, 2007); land tenure arrangements that provide more security to those who depend on rented land (Adjei-Nsiah et al., 2007); logics and procedures for agenda setting in science that are able to capture issues that marginalised groups find important (Leeuwis et al., 2017); places and modes for conducting research that guarantee inclusion of women and youth (Meinzen-Dick et al., 2011); delivery of services like extension and credit to those who are not considered viable by private sector parties (Feder et al., 2011); arrangements for distributing a greater share of benefits along the value chain to farmers (McMichael, 2013); and creation of labour opportunities in and outside agriculture (Giller, 2020). Ignoring such social and institutional issues reflects an over-focus on technology and easily obscures the political character of interventions. In response to the dominant narrative of progress and growth, there are examples of more ‘alternative’ interventions that try to actively change the dominant market-led regime. For example, grassroot movements like Via Campesina and Alliance for Food Sovereignty in Africa (AFSA) start from ecological principles of community and environmental sustainability (Boogaard, 2019; Da Costa & Mcmichael, 2007; McMichael, 2016). These interventions can be seen as niche innovations that come from outside the dominant regime. However, these interventions have a hard time to break through the existing regime because they either hit hard opposition from stakeholders that support the existing regime and stay marginal, or because they are somehow absorbed and subsequently ‘neutralized’ by dominant regime players when they institutionalize (see e.G. Rossi, 2017; Rossi et al., 2019; Schiller et al., 2019).

In addition, it has been argued that planned interventions generally approach change and development as a rational process (Leeuwis, 2004; Long & Van der Ploeg, 1989), not as a process of struggle between regime and niches. As such, there tends to be a lack of attention for mechanisms that keep the existing regime in place and for interventions that may destabilize the existing regime, and as a consequence regime issues are often left unaddressed (Kivimaa & Kern, 2016; Pigford et al., 2018). Historians too have noted that “no transition is planned and coordinated “from the outset”” (Geels & Schot, 2007: p402). This brings us back to be earlier discussed feature that food systems do not have a central locus from which change can be steered and engineered (see Section 2).

### International development has become a regime in itself

As we have seen, many development programs seem unable to change the existing regime and as such poverty is reproduced. To make it more complex, the international development sector has become a regime in itself. This regime is characterized by multiple actors like academics, practitioners, governments, non-government organizations, donor organizations, and development researchers who negotiate and form coalitions about how to reduce poverty and increase food security. These actors have different perspectives on poverty, which make it an ambiguous and contested concept (Cobbinah et al., 2013). For example, among researchers there are (at least) five different poverty theories, with different perspectives on what causes poverty, how it should be measured and the type interventions, including: the perspective of an individual theory of poverty, a culture of poverty perspective, a structure of poverty perspective, a geography of poverty perspective, and a cycle of poverty perspective (Bradshaw, 2007). Moreover, theories about poverty may differ from how ‘poor’ people see themselves. Poverty is thus not a neutral concept. Instead, it is socially constructed in the sense that “the definition of poverty and the policies addressing it are all shaped by political biases and values” (Bradshaw, 2007: p9). Poverty is thus a highly political concept and it is important to pay attention to the political character of poverty interventions by making underlying values, principles and rationales explicit.

It goes beyond the scope of the current paper to extensively discuss underlying values and principles of the existing regime of international development (see e.g. Da Costa & Mcmichael, 2007). However, there is one point that deserves particular attention for those aiming at system transformation. In the current regime of international development, ‘the poor’ are considered subject of analysis who are generally located in the Global South, while framing and analysis occurs largely in and by the Global North. As such, poverty and development are fundamentally Eurocentric^4^ constructs (e.g. Ziai & Escobar, 2007). Eurocentrism^5^ can then be seen as a regime constraint that is hampering a transformation in international development. For example, according to Bhattacharyva (2016) successfully developed countries have institutions that operate according to a neo-liberal logic. This view is in line with development interventions that are based on the idea that a neo-liberal and capitalist economy is needed to overcome poverty and ‘underdevelopment’. However, such a view does not address the current regime of international development itself, but instead reinforces a Eurocentric view within the existing regime. As such, the MLP model also draws attention to more philosophical, epistemological and normative questions about the desired direction of change. Acknowledging Eurocentrism in international development also means acknowledging the role the West has played – intended or unintended – in creating and maintaining the current international development regime, including unequal relations between the Global North and Global South. For example, the international loan system has some problematic historical colonial roots (Bujo, 1998). In response to the Covid19 crisis a group of African intellectuals published an open letter, in which they plead “to break with a model of development based on the vicious cycle of indebtedness” (Soyinka et al., 2020).

### To conclude

With the above arguments we showed how regime and niche levels interact, and that poverty interventions had little influence in changing the existing dominant socio-technical regime. The case study underlines how difficult it is to enhance system transformation, and that planned interventions may have rather limited effect as they tend to ignore key political, competitive and institutional dynamics and processes. System transformations are non-linear, long-term, multi-actor processes, that are not very amenable to planning and control, and associated with processes of self-organisation. Based on the above theoretical and historical insights we thus have argued that the possibilities for steering and purposeful (re)design of food systems are limited. In fact, planned interventions are usually directed at only a part of the system and can worsen the situation and lead to unintended outcomes in other spheres, such as increased inequalities. Hence, it is important to recognize limitations to intervene in a system due to robustness or resilience of the dominant regime. Does this mean that nothing can be done at all? No, that is not our argument. Systems always change over time and people play an active role in this, which suggests that intervention can indeed matter (Klerkx & Rose, 2020). However, it does mean that there are specific considerations that should be taken into account when designing interventions. In the next section we point to key strategies and principles that may be used to influence food system transformation.

## Implications for governing food system transformation & food system synthesis

We started this paper with describing several important features to consider when thinking about food system transformation. We saw that interaction between people is central, and that different actors have different views, set different priorities, and may make different choices between trade-offs. This means that in processes of negotiation and dialogue between stakeholders, discussions about trade-offs will inherently come to the fore. Although system analysis can generate detailed knowledge and understanding about food system dynamics and the likely positive or negative consequences and trade-offs of alternative courses of intervention, it will not result in mutually supported decisions. In the end, humans will make the decisions based on practical, political, economic, normative, and ethical considerations. *Food system synthesis* emphasizes this governance aspect of food system transformation, in which explicit attention is paid to decision making processes and their legitimation in transformation processes, thus: how decisions are made, who decides, who is in- or excluded, what power inequalities are at play, and what – or whose – knowledge is in- and excluded.

These questions are all the more important in ‘development’ contexts which are often characterized by deep unequal power balances and the need to overcome post-colonial legacies in steering system transformation. A useful framework when designing interventions in food system transformation may be that of Responsible Innovation, because it reveals important questions with regard to responsible and just decision making, which often remain implicit. The framework consists of four elements that should be taken into account when designing innovations: (A) anticipation of protentional positive and negative consequences of the innovation, (I) inclusion of all affected parties and viewpoints, (R) reflexivity on values and assumption underlying design choices (R) responsiveness to changing societal demands and concerns (Ludwig & Macnaghten, 2020; Stilgoe et al., 2013). Based on insights from the AIRR framework and the MLP model, we provide seven implications for responsible and just governance of food system transformation. Governance in this sense, is approached from the perspective of change processes based on system innovation literature. We thus look at *governing transformation*, which is different from governance of value chains. The latter refers to governance frameworks of global commodity chains (GCC), global value chains (GVC), and global production networks (GPN) that study the governance of, in or through value chains (see e.g. Bush et al., 2015). Instead, governance of transformation processes looks at types of interventions that are needed to make systems change.

When faced with the ambition to transform a complex multi-facetted system, a question that immediately arises is ‘how and where to start?’. In relation to this, it is important to highlight two starting points. First of all, it is clear that one cannot change a complex system by intervening at a single point in the system (i.e. through a specific policy or technology intervention) since there are too many interdependencies and dimensions involved that cannot be tackled by a single intervention. At the same time, it is neither realistic to approach a complex system in its entirety, and intervene at every possible level, dimension and location at the same time. Such an approach would be practically unfeasible and paralyzing (in terms of resources, funds and knowledge required), and also ignore the insight that systems cannot be designed, engineered and controlled in any detail. Thus, we need to think in terms of a still overseeable set of interventions that are likely to resonate with each other, and that also accommodate or reinforce desirable directions of self-organisation and emergence. Through its emphasis on the interplay between niche, regime and landscape level, the MLP model offers some guidance for thinking about a coherent set of interventions. Below, we elaborate seven governance strategies that we recommend to policy-makers: (1) Creating and supporting variation, (2) Capturing and supporting existing diversity, (3) Temporary protection of niche-level initiatives, (4) Analysis of landscape trends and visioning, (5) Fostering landscape level pressures and active regime destabilization, (6) Identifying plausible leverage point, (7) Process investment in coalition building, collaborative research and media presence.

As will become clear below, the recommendations are linked to finding, creating and supporting specific entry points for change at the level of niche, regime and landscape. For example, what can be done to find and support niche innovations so that these can break through or change the dominant socio-technical regime? Such a governance perspective may lead to a multi-dimensional set of interventions that are eventually aimed at building and shifting societal discourse coalitions. It is important to clarify that the governance strategies do not provide a direction of change or choice in a trade-off, because such decisions should be made by the involved actors in a process of dialogue and negotiation. Instead, the strategies address what can be done to enhance food system transformation as well as how this can be done – illustrated with several concrete examples.

### Creating and supporting variation

In order to be able to respond to emerging challenges, pressures and opportunities it is important that there exists a diversity of initiatives for change at niche level (Geels & Schot, 2007). We cannot design the future in any detail, but we can ensure that sufficient technological and institutional innovations are explored, developed and experimented with under realistic conditions to increase the chances that some of them become sufficiently mature and competitive to alter the dominant food system regime. The emergence of new initiatives may be facilitated by the provision of funding opportunities and complementary innovation brokering activities in which new alliances and networks are formed and supported around interesting options (Kilelu et al., 2011; Klerkx et al., 2009). If there is a challenge with water availability for food production due to climate change, for example, networks of stakeholders in society may be supported in developing, testing and piloting a range of options of a technological nature (e.g. water harvesting, increasing organic matter, agro-foresty solutions, irrigation, bunds, etc.) and of a non-technological nature (new legislation, watershed management, water pricing, etc.). Even if most initiatives may fail to build a strong coalition around such options in the short term, the redundancy and learnings are critical to making progress.

In the spirit of responsible governance, it is important that researchers and others involved in piloting a variation of options pay attention to potential positive and negative consequences for different actors and interests. This includes evaluation of whether the experimented innovations are likely to challenge or reproduce the existing regime. There exist a variety of anticipatory methods that can be usefully applied for such purposes together with the stakeholders involved (see Macnaghten, 2016; Macnaghten et al., 2014).

### Capturing and supporting existing diversity

Societal stakeholders are not waiting for scientists and other external parties to take initiative and respond to changing conditions. In addition to the deliberate creation of variation, it may be very useful to investigate what people are already doing, and what diversity already exists at niche level. This may well include efforts to identify and understand the ‘positive deviants’ (Goldstein et al., 2010); that is, those practices or initiatives that seem to be doing better than others in similar conditions (Geels & Schot, 2007). In relation to the water availability example, one may search for farmers, communities or regions seem to be doing relatively well despite serious constraints, and study the underlying mechanisms and solutions. Subsequently, identified initiatives and options may be offered tailor-made forms of support to develop further.

### Temporary protection of niche-level initiatives

In order to become mature and competitive, niche-level initiatives need space to experiment and learn from mistakes (Geels, 2004). This may require various forms of temporary ‘protection’ in the form of funding to cover initial investments, insurance to deal with unanticipated risks and losses and/or permission to ignore certain rules and standards. For example, farmers and communities that are willing to experiment with agro-forestry systems may need to be compensated if the experiment fails, and be allowed to plant and own trees in regions where legal constraints exist. At the same time, it is important to prevent forms of ‘overprotection’ whereby initiatives operate under completely artificial and unrealistic conditions, so that they collapse as soon as protection is lifted (Hommels et al., 2007; Smith & Raven, 2012). Thus, protection offered must be intelligent and incorporate sustainability considerations, and build on realistic assumptions regarding available capacities, infrastructures, resources and motivations.

### Analysis of landscape trends and visioning

In order to get some grip on what niches-level initiatives to support, it is important to get insight in relevant trends and developments at the landscape level (Schot & Geels, 2008). Coinciding trends and developments can pose pressures on the dominant food system regime, and at the same time offer opportunities to come closer to a ‘tipping point’ in system dynamics. A gradual change in consumer preferences towards plant rather than animal proteins, combined with changes in climate and rainfall patterns -for example- may pose challenges to livestock production in a given region and at the same time foster opportunities for intensifying the production of certain crops. In addition, shocks such as COVID 19 may hamper international transport of export crops and import products, and at the same time allow locally processed food products to strengthen their position in urban markets (Van der Ploeg, 2020). Thus, due to the current Covid19 pandemic and accompanying lockdowns across the globe, international food value chains are seen as ‘broken’ or locked, while clearly there is still a societal demand for food (Lecoutere et al., 2020). It has been estimated that the Covid19 crisis will lead to severe food shortages across the African continent. Part of responsible governance is being responsive to changing societal demands and concerns. This means that researchers and project partners should be able to adapt the project direction, aims and interventions when societal conditions change. For example, Central questions are then: How are ongoing projects able to be responsive to this pandemic? Are project partners and donors flexible to adapt log-frameworks or envisioned pathways of change?

In order to identify and anticipate such scenarios, it is important to invest in analysis of dynamics at landscape level, and translate these towards food system scenarios and/or visions about the future. A fit with plausible and/or desirable scenarios and visions may then be used as a criterion for creating and supporting niche-level initiatives.

### Fostering landscape level pressures and active regime destabilization

The kind of pressures that emerge from developments at landscape level do not only arise in a semi-autonomous and self-organised fashion, but can - in the longer term - also be strategically influenced (or perhaps mimicked) through policy and/or social movements. International authorities may, for example, create fertile ground for the introduction of policy measures, taxes and/or deadlines as a means of creating gradual or sudden shifts in pressure. And social movements and advocacy campaigns may in turn put pressure on authorities to indeed introduce and effectuate such measures. For example, health organisations may advocate and lobby for the introduction of a sugar tax and/or labelling system that may help to combat the obesogenic food system in northern countries (Falbe et al., 2016). Thus, organisations that work towards food system transformation may usefully engage in advocacy campaigns aimed at fostering particular pressures, which can at the same time be seen as an effort to undermine specific aspects of the dominant food system regime.

The case study on poverty illustrated that international development has become a regime in itself and it is increasingly acknowledged that concepts like ‘development’ and ‘poverty’ are Eurocentric constructs. A certain destabilization may be needed to change this regime, which can come from inside as well as outside the regime. Taking this seriously as part of responsible governance requires critical self-reflexivity of researchers and project partners^6^ working on food system transformation, which implies asking crucial questions like: Who defines what ‘poverty’ and ‘development’ entails? What are the main underlying assumptions? On what values and epistemologies are definitions, concepts and desired transformation pathways based? To responsibly address and deal with Eurocentrism in intervention design processes, we propose to be more open to values and philosophies that are currently not part of the existing dominant regime. For example, African philosophies have been largely ignored in international development, whereas African values and perspectives are highly relevant for sustainable agricultural development and may provide a counter-hegemonic view towards the dominant capitalist and neo-liberal food regime (Boogaard, 2019).

### Identifying plausible leverage points

The above recommendations are linked to finding, creating and supporting specific entry points for change at the level of niche, regime and landscape. Even if it is clear that -in order to create sufficient variation- a certain amount of redundancy is needed in terms of initiatives and interventions, limitations in funding and capacity usually necessitate selection of entry points that are supported. It is therefore important to think in terms of leverage points (Meadows, 1999, 2009); that is, of entry points in the system (e.g. in the form of constraining or enabling policies, rules, meanings, technologies, communities, stakeholders) where change is most likely to catalyse subsequent self-organizing changes elsewhere in the system. Such catalytic capacity may be rooted in various mechanisms (e.g. power relations, interdependencies, causal links, stakeholder rationales, attractiveness, latent needs, connectedness, etc.) and there is no fixed recipe for finding them, even if there exist analytical strategies (e.g. Klein Woolthuis et al., 2005; Sartas et al., 2020). In any case, identifying plausible leverage points then is likely to require a thorough interdisciplinary understanding of the way in which phenomena at the level of niche, regime and landscape interact with each other, as well as transdisciplinary deliberation with societal agents. In several instances, reward systems have been show to serve as a leverage for other changes. The creation of guaranteed prices for agricultural products in Europe after the Second World War, for example, has arguably catalysed numerous other changes (e.g. in farmer attitudes, the willingness of banks to provide loans, investment in R&D, land reclamation, etc.) that eventually gave rise to a radical change in the European (and global) food system. Eventually, this led not only to abundant food supply and reasonable incomes for farmers in Europe, but also to overproduction, dumping of produce in developing countries, environmental degradation and a technological and economic treadmill which has forced large numbers of farmers to leave agriculture. A relevant question from the perspective of responsible innovation and governance is whether such negative consequences have or could have been foreseen.

### Process investment in coalition building, collaborative research and media presence

A more overarching strategy for catalysing food system transformation is the investment in stakeholder processes in support of the above-mentioned strategies and entry points. Eventually, transformation requires the emergence of a strong coalition for change around promising initiatives, characterised by common goals, a shared discourse and joint strategy (Leeuwis & Aarts, 2011). More often than not, this involves the bringing together of parties and stakeholders who have not collaborated before, and who may have widely diverging interests and worldviews. Aligning interdependent stakeholders around an overlapping vision for the future is not an easy process. It requires that stakeholders learn about each other’s perspectives and about interdependencies in the system, and develop conducive relations and trust. Facilitated interaction, articulation of knowledge demands and joint research and fact finding to address uncertainties and gaps in understanding are known to be important strategies for developing common ground (Leeuwis, 2004; Pruitt & Carnevale, 1993). In addition, food system transformation (or synthesis) requires that interdependent stakeholders settle emerging tensions and diverging interests through integrative forms of negotiation (Aarts & Van Woerkum, 2002; Susskind & Cruikshank, 1987). Such processes of negotiation, knowledge co-creation, and dialoguing between different stakeholders, may be facilitated by bringing stakeholders together in a multi-stakeholder platform – often referred to as innovation platforms (see Textbox 2). Such platforms may usefully operate at both niche and regime level, and pay special attention to collaborative research as a vehicle for developing and testing alternative options, as well as for discovering common starting points and common ground (Hounkonnou et al., 2018). Over the past years, many practical guidelines have been published on how to organize such platforms and develop constructive negotiations and dialogues (see e.g. Boogaard et al., 2013; Pali & Swaans, 2013; Schut et al., 2017). Such practical guidelines may be helpful when designing multi-stakeholder platforms, but they do not answer questions with regard to what and whose niches are to be supported for what purpose and in what direction, and neither do they offer guidance on how set agenda and define activities within a niche.

**Table:** Box 2 Pitfalls and opportunities of multi-stakeholder platforms

There is an increased interest in multi-stakeholder platforms as a governance model for stimulating innovation and development in food systems (Boogaard et al., 2013; Schut et al., 2019) and much research has been done on multi-stakeholder platforms over the past years (see e.g. Kilelu et al., 2013; Schut et al., 2015, 2019; Swaans et al., 2014). There are good grounds for bringing stakeholders together in platforms, but there are also many pitfalls that need to be overcome in order to render them useful for system transformation processes. Without being extensive, we briefly mention a few.	
Several opportunities:	
• Bring interdependent actors together to create meaningful change	
• Come to some kind of coordination, agreement and mutual expectation between platform members	
• Offer space for communication, learning and dispute resolution	
• Jointly define challenges, opportunities and possible solutions and actions	
• Provide access to research capacity and jointly identify research questions	
Several pitfalls:	
• Multi-stakeholder processes are characterized by a certain messiness, tension and competition	
• Platform members tend to disagree on the direction ‘development’ should take	
• Researchers become involved in politics, ethics and legitimacy issues	
• Multi-stakeholder platforms can be hijacked by formal programs	
• It is notoriously difficult to elicit relevant research questions	
• Platforms may be used for diffusion purposes only, failing to address important social, technical and epistemological constraints.	

Here the AIRR framework is helpful, as responsible governance entails that all affected parties and viewpoints should be included in the transformation process. The ‘all-affected principle’ means that justice should be done to those who are affected in terms of redistribution, representation, and recognition (Fraser, 2007; Ludwig & Macnaghten, 2020). The case study on poverty showed that altering regime reconfiguration is inherently difficult because marginalized people and their knowledge remain easily excluded (Lam et al., 2020). Instead, interventions that strengthen the existing regime reinforce unequal power imbalances. Central questions in multi-stakeholder governance are then: Who is in- and excluded in transformation processes? Who decides who is involved? How are decisions made? And is it desirable to include regime actors, to avoid that the process becomes hijacked? These questions not only account for the actors, but also for their epistemologies: Whose knowledge counts? What if knowledge forms in niches are different from the types of knowledge in dominant regimes? Who decides what knowledge is in- or excluded? This calls for epistemic awareness in transformation processes, which means the “awareness that epistemology is an issue and that, therefore, choices can be made about how to think about the issues that arise in any situation” (Armson, 2011: p313). Awareness of diverse epistemologies, however, does not in itself lead to knowledge integration. Instead, there may be partial overlaps as well as gaps between different knowledge systems, such as indigenous knowledge and conventional Western science (Ludwig & El-Hani, 2020).

Eventually, the idea is that the kind of governance interventions indicated above can resonate with each other, and induce the emergence of a strong coalition for change that competes successfully with the incumbent food system regime. An important element and early indicator of coalition formation is the emergence of novel ways of talking about the food system in both policy networks and in society at large. This is because societal change is prepared in human interaction and communication, and becomes visible through ‘discourse coalitions’ (Hajer & Laws, 2006) and shifting conversations (Ford, 1999). When citizens, media, bureaucrats and politicians start to discuss the problems, causes and solutions in food systems differently in their formal and informal conversations, then it is likely that change is in-the-making (Leeuwis, 2013; Leeuwis & Aarts, 2011). In order to resonate in societal conversations, it is important that new problem definitions, options and scenarios are captured and integrated in persuasive storylines rather than only in rational arguments or scientific publications (Van der Stoep, 2014; Van der Stoep et al., 2017). A final recommendation to those who want to stimulate food system transformation, therefore, is to pay deliberate attention to developing well-grounded and at the same time appealing storylines for relevant audiences, and ensure that these are somehow circulated in both conventional mass-media (radio, newspapers, television, etc.) as well as in more informal media spaces (Facebook, Twitter, etc).

## Conclusions

In this paper we argued that food systems are best looked at as complex multi-dimensional systems, which require a process of *food system synthesis*; that is, a transformation process in which food systems are reconfigured to produce more desirable outcomes. Food system synthesis goes beyond food system analysis, as the latter is often geared mainly towards understanding (parts of) the system, and using this understanding to propose options in order to optimise the system through some kind of engineering logic. Such an optimisation approach reflects illusionary assumptions regarding the possibility of steering and controlling transformation, and largely ignores that transformation is -in actual practice- a contested, competitive and political process and not a matter of rational design. Instead, in a process of food system synthesis, interdependent stakeholders need -to some degree- resolve their differences, build conducive relationships and overlapping visions on the future. Food system synthesis or transformation can be seen as an effort to alter undesired emergent properties of the system – such as environmental degradation, economic exploitation, malnutrition, food insecurity, increased inequalities, and poverty – into desired properties such as ‘healthy nutrition’, ‘food security’, ‘wealth’ and ‘environmental sustainability’. Building on the MLP model for understanding how system transformations occur, we suggest that such system change involves non-linear, long-term, multi-actor processes with struggles and tensions between actors operating at regime and niche level. The case study on the persistence of poverty illustrated that the possibilities for steering and purposeful (re)design of food systems are limited, and that interventions can even worsen the situation and lead to unintended outcomes, such as increased inequalities. This, however, does not mean that transformation processes cannot be influenced, but rather that there are specific governance implications that should be taken into account. The framework of responsible innovation can then be helpful in reflecting on where to go and how to adjust. Combining the MLP model with insights regarding responsible innovation we suggest seven governance strategies that policy-makers may usefully apply: (1) Creating and supporting variation, (2) Capturing and supporting existing diversity, (3) Temporary protection of niche-level initiatives, (4) Analysis of landscape trends and visioning, (5) Fostering landscape level pressures and active regime destabilization, (6) Identifying plausible leverage point, and (7) Process investment in coalition building, collaborative research and media presence. Adopting such recommendations would imply a considerable re-orientation of investments in food system transformation, with greater attention to dealing with social, institutional and political dimensions of innovation and transformation. This simultaneously implies investment in processes with uncertain outcomes, and the need to develop novel and credible ways of assessing progress in long-term transformation processes. Finally, policy makers may need to rethink their role in supporting processes of food system transformation. While in some situations they may be in a powerful position to contribute to changes in socio-technical regimes (e.g. by changing laws, regulations and incentive structures) they also need to consider that they may well be part of the problem and play a prominent role in reproducing undesirable system outcomes. In those cases, the governance interventions proposed can be seen as strategies to help bring about countervailing power and opposition under the guise of innovation policy.

## Footnotes

1 We recognize the wide variety in definitions and approaches towards poverty, e.g. including absolute poverty, relative poverty, food poverty, nutrition poverty. In this paper ‘poverty’ is not restricted to the monetary indicator of the poverty line of 2USD per day, but it is broader: it refers to structural, stagnating – or even increasing - inequalities of poor, marginalized, and exploited groups of people with regard to e.g. health, education, basic needs, their living environment, rights, and gender relations (Cobbinah et al., 2013; Green & Hulme, 2005).

2 It is important to note the difference between ‘regime’ and ‘system’: “System then refers to tangible and measurable elements (such as artefacts, market shares, infrastructure, regulations, consumption patterns, public opinion), whereas regimes refer to intangible and underlying deep structures (such as engineering beliefs, heuristics, rules of thumb, routines, standardized ways of doing things, policy paradigms, visions, promises, social expectations and norms). So ‘regime’ is an interpretive analytical concept that invites the analyst to investigate what lies underneath the activities of actors who reproduce system elements.” (Geels, 2011)

3 In this case study we focus on poverty interventions in the Global South, which are largely led by the Global North. However, we clearly recognize that poverty is a global problem. The mainly one-way focus of the Global North towards poverty in the Global South is not unproblematic. We will therefore return to this point towards the end of this paper when we address more philosophical and normative questions.

4 The Eurocentric concept of development refers to labelling Western Europe and North America as ‘developed’ and Africa, Asia and Latin America as ‘underdeveloped’, in which “the own society is perceived as constituting an ideal norm and other societies are perceived as imperfect deviations from this norm, as inferior versions of the self, which are, however, in the process of approaching the norm – although they will never reach it.” (Ziai & Escobar, 2007: p8).

5 It should be noted that the MLP model has been developed on the basis of historical case studies in a Western context, which means that there is an inherent risk of reproducing Eurocentrism when applying the MLP model to poverty contexts (Ramos-Mejía et al., 2018).

6 This also counts for at least two authors of the current paper (CL and BB), who both have a Western educational and cultural background. Moreover, both the AIRR Framework as well as the MLP model are developed in a Western context. Hence, one has to be careful when using these frameworks in other cultural contexts.
